# A quantitative PCR approach for quantification of functional genes involved in the degradation of polycyclic aromatic hydrocarbons in contaminated soils

**DOI:** 10.1016/j.mex.2016.02.005

**Published:** 2016-03-09

**Authors:** Esmaeil Shahsavari, Arturo Aburto-Medina, Mohamed Taha, Andrew S. Ball

**Affiliations:** a Centre for Environmental Sustainability and Remediation, School of Science, RMIT University, Bundoora, Victoria 3083, Australia; b Instituto Tecnológico y de Estudios Superiores de Monterrey (ITESM), 72800 Puebla, Mexico; c Department of Biochemistry, Faculty of Agriculture, Benha University, Moshtohor, Toukh 13736, Egypt

**Keywords:** Bioremediation, Hydrocarbons, PAHs, PCR, qPCR, Functional genes

## Abstract

Polycyclic aromatic hydrocarbons (PAHs) are major pollutants globally and due to their carcinogenic and mutagenic properties their clean-up is paramount. Bioremediation or using PAH degrading microorganisms (mainly bacteria) to degrade the pollutants represents cheap, effective methods. These PAH degraders harbor functional genes which help microorganisms use PAHs as source of food and energy. Most probable number (MPN) and plate counting methods are widely used for counting PAHs degraders; however, as culture based methods only count a small fraction (<1%) of microorganisms capable of carrying out PAH degradation, the use of culture-independent methodologies is desirable.

• This protocol presents a robust, rapid and sensitive qPCR method for the quantification of the functional genes involved in the degradation of PAHs in soil samples.
• This protocol enables us to screen a vast number of PAH contaminated soil samples in few hours.
• This protocol provides valuable information about the natural attenuation potential of contaminated soil and can be used to monitor the bioremediation process.

## Graphical abstract

**Figure:**
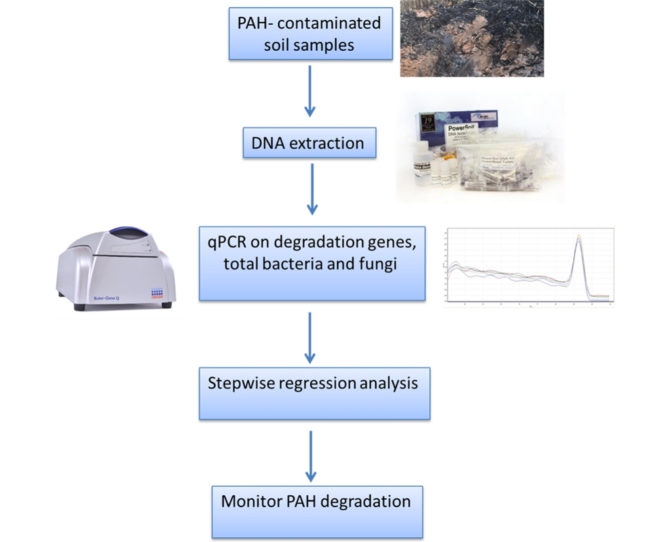

## Method details

### Protocol background

Oil pollution is a major problem globally as a result of our great dependency on hydrocarbons and hydrocarbon by-products. Oil pollution can occur on a large scale such as ocean and land spills, with devastating effects on macro- and micro-organisms living in these environments. Oil contains thousands of compounds and some of them such as polycyclic aromatic hydrocarbons (PAHs) are toxic and carcinogenic [1], [2]. Bioremediation or the use of microbes (mainly bacteria and fungi) represents a promising, cheap approach to the degradation or clean up of PAH-polluted environments.

Bacterial hydrocarbon degraders harbor functional genes that make them capable of degrading a range of different hydrocarbon compounds. Targeting and measuring functional genes involved in hydrocarbon degradation reflects the potential of a microbial community to degrade a contaminant such as PAHs in polluted soil [3], [4], [5]. Recent developments in the application of molecular tools such as DGGE and TRFLP to environmental microbiology have aided in the identification of the microbes responsible for the hydrocarbon degradation. However, these methods cannot be used for quantification.

Real-time PCR or quantitative PCR (q-PCR) is a very sensitive technique that allows the quantification of a specific nucleic acid sequence in real-time through PCR product detection. The detection of PCR products in real time is achieved by measuring the fluorescence produced during the extension step. Fluorescence is measured in each cycle and it is proportional to the amount of PCR product. Thus, the accurate quantification of the initial concentrations of DNA, cDNA and RNA can be achieved. There are two alternatives to produce fluorescence and these involve the use of (i) probes that are sequence-specific and fluorescently labeled or (ii) fluorescent dyes that bind to double-stranded DNA (ds DNA).

SYBR Green is a fluorescent dye that binds to ds DNA via intercalation between adjacent pairs and represents one of the most widely used fluorescent dyes. It emits a fluorescent signal while binding and can be used with any real-time cycler since its excitation and emission maxima are at 494 and 521 nm respectively. As mentioned above, the signal intensity is proportional to the accumulation of PCR product but primer-dimers and non-specific products will also add to the signal. Thus, primers need to be highly specific when using SYBR Green to avoid overestimation of the target.

In the present work, an efficient, cheap and reproducible qPCR method that uses SYBR Green is presented which helps researchers quantify hydrocarbon-degrading genes in bacteria and in turn monitor bioremediation of PAHs-polluted sites.

As a preliminary step to the work reported here, we tested various primer pairs and selected those in Table 1 as biomarkers to monitor the degradation of PAHs in both laboratory and field.

### DNA extraction

We recommend MoBio Powersoil^®^ DNA Isolation Kit (MoBio) for DNA extraction from contaminated soil. In terms of the protocol:

• Weigh 0.25–0.5 g of soil into the bead solution tube (as per the manufacturer's instructions).
• Lyse the cells by adding the sample to the lysing matrix tubes and homogenize for 2 × 20 s in a FastPrep (MP Biomedicals) homogenizer.
• Proceed following the manufacturer's instructions.
• Check the quality and quantity of DNA using 1% agarose gel electrophoresis or a NANODROP. In the case of using a NANODROP, the ratio of absorbance at 260 nm and 280 nm is used to evaluate the purity of DNA. A ratio between 1.8–2.2 would be acceptable for qPCR analysis.

### Primers used for qPCR

A list of primer sets used for qPCR is shown in Table 1. These primers were selected based on their wide coverage in terms of bacterial PAH catabolic genes. Furthermore, primer design can also be carried out based on the sequence of genes of interest in GeneBank. Free platforms such as OligoArchitect™ Primer (Sigma) or Primer-BLAST (NCBI) can be used for this purpose.

### Preparation of standard curve and samples

The absolute quantification in the qPCR method is based on the quantification of standards. In general, three types of standards can be used; (i) cleaned PCR products of the gene of interest, (ii) cleaned PCR products of the gene of interest cloned into a plasmid, and (iii) the total genome of bacteria harboring the gene of interest (assuming that the genome contains one copy per genome).

All these standards provide satisfactory results; however we recommend using cleaned PCR products of the gene of interest as this standard does not require any cloning steps or bacterial isolation process.

Serial dilutions (10^−1^ to 10^−8^) of cleaned PCR products of the gene of interest for the generation of the standard curve are used in this protocol. Standards are used in triplicate for each dilution.

• Perform different dilutions of samples (for example 1:2, 1:5 and 1:10) with sterile nuclease-free water. A previous sample with the highest gene copy numbers should be used for the assessment. Our results show that in most cases, 1:10 dilution is enough for qPCR. It is important to note that new dilutions should be performed each time for qPCR analysis.

### Preparation of a mastermix

**Fig. 1:**
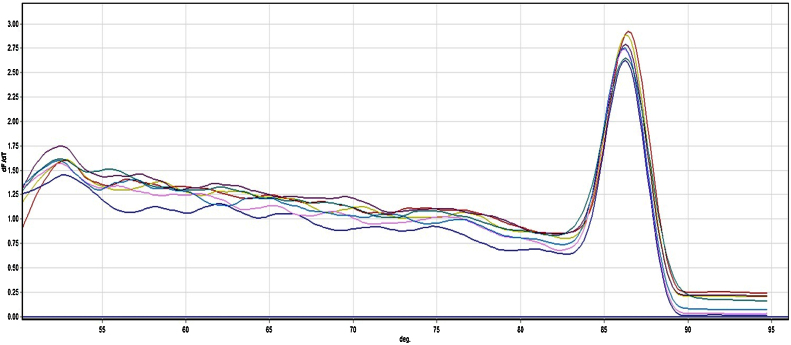
Melt curve analysis of PAH-ring hydroxylating dioxygenase genes from Gram negative bacteria (PAH-RHD GN) for soil samples contaminated with PAHs.

• Prepare a mastermix for each of the genes to amplify. We use KAPA SYBR^®^FAST qPCR mastermix, however other qPCR mastermix's from other suppliers may be used instead.
• Select the genes to amplify from Table 1.
• It is important to note that primer pairs 341F/518R and ITS1F/5.8 s amplify the 16S rDNA and the ITS regions respectively. The quantification of total 16S rDNA and ITS region provides information about the total microbial community dynamics affected by PAHs. In addition, by normalization of the gene copies of a functional gene with 16S rDNA gene copies resulted in a ready comparison of the PAH-degrading populations among the soil samples.
• Add 19 μl of the cocktail (2× KAPA SYBR^®^ FAST qPCR Mastermix + forward and reverse primers + PCR-grade water) to each reaction tube.
• As the last step, add 1 μl of DNA Template.

• Make a mastermix for the desired number of samples to amplify according to Table 2. We used KAPA SYBR^®^FAST qPCR Mastermix; however other qPCR mastermix from other suppliers may be used instead.
  Ideally, all primer pairs should be tested in order to fully monitor PAHs degradation. Selection of the primers depends on the contaminant type, budgets and time. However it is recommended that at least two sets of primers together with total bacteria and fungi primers should be used. We recommend the use of ring hydroxylating dioxygenase genes from Gram-negative and -positive bacteria (PAH-RHD GNF/R & PAH-RHD GPF/R) as these primer sets amplify various genes involved in the degradation of PAHs [6]. It is important to note that primer pairs 341F/518R and ITS1F/5.8 s amplify the 16S rDNA and the ITS regions respectively.
  We use a QIAGEN Rotor-Gene system for qPCR analysis; other system from different companies such as IQ5 (Biorad) or 7300 Real-Time PCR System (Applied Biosystems) can be used, however some optimizations should be considered.
• Run the PCR program according to the primer conditions as shown in Table 2. qPCR cycling conditions are an initial denaturation step at 95 °C (5 min) followed by 40 cycles of 95 °C denaturation (10 s), annealing at different temperature for each primer set according to Table 1 (30 s), 72 °C extension (30 s), 80 °C primer dimer removal and signal acquisition (10 s).
  Due to the formation of primer dimers, we recommend that signal acquisition should be carried out at 78–80 °C. In this temperature range, primer dimers will be removed. However, melt curve analysis should be carried out to detect the presence of primer-dimers. This step is carried out between 55 and 95 °C with 0.5 °C increments every 5 s. An example of a melt cure is presented in Fig. 1. Alternatively, qPCR amplification products can be run using agarose gel electrophoresis to check for primer dimers and non-specific bands.

### Data analysis

• Check the linearity of the standard curves and PCR efficiency
  The standard curve is generated by plotting CT values against the logarithm of the input amount of the standard material and it is usually created by performing at least five dilutions of standards (clean PCR product) of known concentration. Linear standard curves with *r*^2^ > 0.95 (ideally >0.99) are considered good standard curves. In addition, PCR efficiency must be calculated based on the following formula: PCR efficiency = 10^(−1/Slope)^ − 1 where the slope is given by the slope of the standard curve. This is because the slope provides an indication of the efficiency of the quantitative PCR. A slope of −3.322 indicates that the amount of PCR product doubles in each cycle, representing a PCR efficiency of 1 or 100%. PCR efficiency between 0.9–1.1 is acceptable for this protocol.
• Calculate the number of gene copies according to the following formula:
  $$Y\;\text{molecules/}\mu \text{l}=(X\;\text{g/}\mu \text{l DNA/}[\text{PCR product length in base pairs}\times 660])\times 6.022\times 10^{23}\text{.}$$
• Express the gene copies as log_10_ of gene copy numbers per g dry soil.
  Assessment of functional genes in conjunction with total bacteria (341F/518 R) and fungi (ITS1F/5.8 s) gives valuable information regarding the potential of the microbial communities to degrade PAHs. The functional genes can also be used as biomarkers to monitor the bioremediation. Our data showed that when ring hydroxylating dioxygenase genes from Gram-negative and -positive bacteria (PAH-RHD GNF/R & PAH-RHD GPF/R) are above 10^4^ and 10^6^ respectively (per g dry weight of soil), bioremediation most likely will be successful (data no shown).

An example of gene copies detected in a PAH contaminated soil is shown in Table 3. The gene copies were obtained from two different PAH contaminated soils with different level of PAHs. The results showed that a significant increase in hydroxylating dioxygenase gene copies in Gram-positive bacteria in both soils was observed after 12 weeks of bioremediation (in this case using biostimulation), confirming their contribution to the degradation of PAHs. In contrast, the number of copies of the pyrene degrading gene (*nidA*) decreased from 6.64 log gene copies/g dry soil at Week 0 to 2.54 log gene copies/g dry soil at Week 12 in Soil B compared to little change in Soil A (Table 3), suggesting that bacteria harboring the *nidA* gene may have had only a limited impact on the degradation of PAHs in soil B. Furthermore, the numbers for total bacteria and fungi remain constant throughout the 12 weeks suggesting a stable microbial community during the hydrocarbons degradation.

Apart from the usual statistical analysis such as ANOVA, we highly recommend the use of regression analysis (e.g. backward stepwise regression) using statistical software such as SPSS or XLSTAT to identify any relationship between the degradation rate of PAHs and gene copies numbers. The bioremediation equation is *Y* = *aX* + *bY* + *cZ* + … + *C*, where *Y* is the concentration of PAHs at certain time point; *X*, *Y* and *Z* are gene copies of genes of interest which showed significant effect in the regression model; *a*, *b*, *c* are regression coefficients and *C* is the intercept.

## Figures and Tables

**Table 1:** Primer sets targeting PAH-degrading, 16S rDNA and ITS region genes used for this protocol.

Primer name	Target gene	Annealing temperature (°C)	Sequence (5′–3′)	Reference
NAH-F/NAH-R	Naphthalene dioxygenase	47	F: CAAAA(A/G)CACCTGATT(C/T)ATGG R: A(C/T)(A/G)CG(A/G)G(C/G)GACTTCTTTCAA	[7]
NidA-F/NidA-R	Pyrene dioxygenase genes	59	F: TTCCCGAGTACGAGGGATAC R: TCACGTTGATGAACGACAAA	[8], [9]
C23O-F/C23O-R	Catechol-2,3-dioxygenase genes	58	F: AAGAGGCATGGGGGCGCACCGGTTCGATCA R: CCAGCAAACACCTCGTTGCGGTTGCC	[10]
CATA-F/CATA-R	Catechol-1,2-dioxygenase genes	58	F: ACVCCVCGHACCATYGAAGG R: CGSGTNGCAWANGCAAAGT	[11]
PAH-RHD GNF/R	PAH-ring hydroxylating dioxygenase genes from Gram negative bacteria	57	F: GAGATGCATACCACGTKGGTTGGA R: AGCTGTTGTTCGGGAAGAYWGTGCMGTT	[6]
PAH-RHD GPF/R	PAH-ring hydroxylating dioxygenase genes from Gram positive bacteria	54	F: CGGCGCCGACAAYTTYGTNGG R: GGGGAACACGGTGCCRTGDATRAA	[6]
341F/518R	16S rDNA	55	F: CCTACGGGAGGCAGCAG R: ATTACCGCGGCTGCTGG	[12]
ITS1F/5.8 s	ITS region	53	F: CTTGGTCATTTAGAGGAAGTAA R:CGCTGCGTTCTTCATCG	[13]

**Table 2:** qPCR cocktail's components.

Cocktail's components + DNA template	20 μl rxn	Final conc.
2× KAPA SYBR^®^ FAST qPCR Master Mix Universal	10 μl	1×
Forward primer (10 pM)	0.4 μl	200 nM
Reverse primer (10 pM)	0.4 μl	200 nM
PCR-grade water	8.2 μl	N/A
Template DNA	1 μl	<20 ng μl^−1^

**Table 3:** Gene copy numbers of interest genes at Week 0 and Week 12 in 2 different contaminated soils during bioremediation.

Soil		Gene copies (log gene copies/g dry soil)			Total bacteria	Total fungi
		*nida*	PAH-RHDa GN	PAH-RHDa GP		
Soil A	Week 0	7.20	7.18	2.01	9.79	7.07
	Week 12	7.34	6.57	4.16	9.16	6.31

Soil B	Week 0	6.64	6.36	3.97	10.37	6.76
	Week 12	2.54	6.75	6.67	10.5	7.54

## References

[1] Shahsavari E., Adetutu E.M., Anderson P.A., Ball A.S. (2013). Necrophytoremediation of phenanthrene and pyrene in contaminated soil. J. Environ. Manage..

[2] Blyth W., Shahsavari E., Morrison P.D., Ball A.S. (2015). Biosurfactant from red ash trees enhances the bioremediation of PAH contaminated soil at a former gasworks site. J. Environ. Manage..

[3] Yang Y., Wang J., Liao J., Xie S., Huang Y. (2015). Abundance and diversity of soil petroleum hydrocarbon-degrading microbial communities in oil exploring areas. Appl. Microbiol. Biotechnol..

[4] Adetutu E.M., Ball A.S., Weber J., Aleer S., Dandie C.E., Juhasz A.L. (2012). Impact of bacterial and fungal processes on ^14^C-hexadecane mineralisation in weathered hydrocarbon contaminated soil. Sci. Total Environ..

[5] Shahsavari E., Adetutu E.M., Anderson P.A., Ball A.S. (2013). Plant residues – a low cost, effective bioremediation treatment for petrogenic hydrocarbon-contaminated soil. Sci. Total Environ..

[6] Cébron A., Norini M.-P., Beguiristain T., Leyval C. (2008). Real-time PCR quantification of PAH-ring hydroxylating dioxygenase (PAH-RHDα) genes from Gram positive and Gram negative bacteria in soil and sediment samples. J. Microbiol. Methods.

[7] Baldwin B.R., Nakatsu C.H., Nies L. (2003). Detection and enumeration of aromatic oxygenase genes by multiplex and real-time PCR. Appl. Environ. Microbiol..

[8] Peng J.-J., Cai C., Qiao M., Li H., Zhu Y.-G. (2010). Dynamic changes in functional gene copy numbers and microbial communities during degradation of pyrene in soils. Environ. Pollut..

[9] DeBruyn J.M., Chewning C.S., Sayler G.S. (2007). Comparative quantitative prevalence of *Mycobacteria* and functionally abundant *nidA*, *nahAc*, and *nagAc* dioxygenase genes in coal tar contaminated sediments. Environ. Sci. Technol..

[10] Sei K., Asano K.-I., Tateishi N., Mori K., Ike M., Fujita M. (1999). Design of PCR primers and gene probes for the general detection of bacterial populations capable of degrading aromatic compounds via catechol cleavage pathways. J. Biosci. Bioeng..

[11] El Azhari N., Devers-Lamrani M., Chatagnier G., Rouard N., Martin-Laurent F. (2010). Molecular analysis of the catechol-degrading bacterial community in a coal wasteland heavily contaminated with PAHs. J. Hazard. Mater..

[12] Muyzer G., De Waal E.C., Uitterlinden A.G. (1993). Profiling of complex microbial populations by denaturing gradient gel electrophoresis analysis of polymerase chain reaction-amplified genes coding for 16S rRNA. Appl. Environ. Microbiol..

[13] Fierer N., Jackson J.A., Vilgalys R., Jackson R.B. (2005). Assessment of soil microbial community structure by use of taxon-specific quantitative PCR assays. Appl. Environ. Microbiol..

